# Neuromuscular block in patients 80 years and older: a prospective, controlled study

**DOI:** 10.1186/s12871-021-01443-1

**Published:** 2021-09-13

**Authors:** Denis Schmartz, Raouf Sghaier, Paul Bernard, Jean François Fils, Thomas Fuchs-Buder

**Affiliations:** 1 CHU Brugmann, Université Libre de Bruxelles, 4 Place Van Gehuchten, 1020 Bruxelles, Belgium; 2 CHRU de Nancy, Rue du Morvan, 54511 Vandœuvre-lès-Nancy, France; 3 Ars Statistica, Boulevard des Archers 40, 1400 Nivelles, Belgium; 4 CHRU de Nancy, Université de Lorraine, Rue du Morvan, 54511 Vandœuvre-lès-Nancy, France

**Keywords:** Neuromuscular blocking agents, Geriatric patients, Accelerometry, Neuromuscular monitoring

## Abstract

**Background:**

An increasing number of patients older than 80 years are undergoing anesthesia, but little information is available regarding pharmacodynamic effects of myorelaxants in this population. This study aims to compare the time course of rocuronium neuromuscular block in patients ≥ 80 years with those of younger adults.

**Methods:**

Under total intravenous anesthesia with propofol and sufentanil, time course of a bolus of rocuronium 0.6 mg/kg neuromuscular block was assessed with acceleromyography in patients ≥ 80 and in patients 20–50 years old. Onset time, clinical duration, duration until 90% and 100% recovery of baseline were determined.

**Results:**

Data from 32 patients were analyzed, 16 were ≥ 80 years and 16 were 20–50 years old. Demographic data are shown in Table 1. In the group ≥ 80, onset time was 190 s ± 46 s compared to 123 s ± 40 s in the group 20–50, *P* < 0.001 and the clinical duration was 52 [48–69.5] min and 36 [34–41] min, respectively, *P* < 0.001. Duration to 90% recovery of baseline was 77.5 [71–88.5] min and duration to 100% recovery of baseline was 91.2 [82.2–98] min in patients ≥ 80 years and the corresponding values in the patients 20–50 years old were 53.5 [49–55.5] min and 59.5 [56.5–70.25] min, respectively, *P* < 0.001.

**Conclusion:**

Compared to younger adults rocuronium shifted in patients ≥ 80 years from a rapid onset, intermediate acting compound to a slower onset, long-acting compound.

**Trial registration:**

ClinicalTrials.gov identifier: NCT03551652 (29/05/2018).

## Introduction

Neuromuscular blocking agents (NMBA) are used to facilitate tracheal intubation and improve surgical conditions [1–3]. Residual neuromuscular blockade is commonly seen postoperatively and patients with residual paralysis have an increased risk of postoperative respiratory complications such as hypoxemia and upper airway obstruction [4–6]. The risk of residual paralysis and respiratory complications is markedly increased among elderly patients [7, 8]. The presence of small degrees of muscle weakness after tracheal extubation may significantly affect outcome in this patient population, as the elderly have limited physiologic reserve [9]. In particular, pharyngeal function and muscle strength may be impaired in patients older than 65 years, and the residual effects of NMBA’s may further worsen this impairment [8, 10]. Approximatively 50% of elderly patients will require anesthesia for surgical interventions and increasing age may be associated with greater morbidity and mortality after anesthesia [11, 12]. According to Eurostat’s 2019 edition of Ageing Europe report, the oldest old (i.e. patients ≥ 80 years) are within the group of elderly the fastest growing segment of the population at large. Unfortunately, pharmacodynamic data of NMBA in patients ≥ 80 years old are sparse.

The aim of this prospective, controlled observational study was to compare the time course of neuromuscular blockade after rocuronium 0.6 mg/kg in patients ≥ 80 years old with patients between 20-50 years. We hypothesized that patients ≥ 80 years old have slower onset and increased duration and recovery of neuromuscular blockade.

## Methods

The research protocol was approved by the institutional review committee (Comité de Protection des Personnes EST III, 10 September 2018, referral number 200, chairperson Pr Y. Martinet) and registered at Clinicaltrials.gov (NCT03551652 on 29/05/2018) before enrollment of the first patient. All patients gave written informed consent, and all clinical data were obtained in our department. 35 patients scheduled for a surgical procedure under general anesthesia were included between 15 May 2020 and 16 March 2021. Exclusion criteria were hypersensitivity to rocuronium or any other of the drugs used during this study, contraindication to rocuronium, planned rapid sequence induction (RSI), refusal to participate, and the absence of written informed consent. This study is reported according to the STROBE statement.

Patients were included in one of two groups based on age: an elderly cohort in group 80^+^ (≥ 80 years old) and a younger cohort in group 20–50 (age 20–50 years).

### Anesthesia technique

Monitoring established on arrival in the operating room included electrocardiography, non-invasive arterial pressure, pulse oximetry, and capnography. After preoxygenation, total intravenous anesthesia (TIVA) was induced in all patients with 1.5–2.5 mg/kg propofol and 10 μg sufentanil and maintained with 4–10 mg/kg/h propofol and bolus doses of sufentanil (5–10 μg). Nitrous oxide and halogenated agents were avoided. Neuromuscular block was established with a single dose of rocuronium 0.6 mg/kg without any further injection. The bolus of rocuronium was given over 5 s. By using a warming blanket, the central temperature was maintained over 35 °C and the peripheral body temperature measured at the thenar eminence of the palm was maintained at least at 32° C. End tidal pressure of carbon dioxide was maintained between 32–36 mm Hg.

### Neuromuscular monitoring

Neuromuscular blockade was quantified with TOFscan (IMED, Marseille, France) according to Good Clinical Research Practice (GCRP) in pharmacodynamic studies of neuromuscular blocking agents [13]. This device has an adapter with a built-in three-dimensional acceleration transducer applying a constant preload. The TOFScan hand adapter was fixed on the dominant hand and surface electrodes were placed on the cleaned skin over the corresponding ulnar nerve as described previously [14]. The negative electrode was placed near the wrist and the positive electrode 3 cm proximally and the arm and fingers were secured with velcro straps to prevent movement artifacts during stimulation. Neuromuscular monitoring was started after induction of anesthesia but before administration of rocuronium. The current intensity was fixed at 50 mA. To obtain raw TOF ratio’s the T4/T2 algorithm of the device was inactivated. After some initial TOF stimulation, a 5 s, 50 Hz tetanic stimulation was applied followed by TOF stimulations for 10–15 min until a stable response was achieved—defined as less than 5% variation in the TOF-ratio for at least 2 min. Thereafter the device was set to deliver TOF stimulations at a 15 s interval and the following time intervals were measured as defined: onset time, defined as the time from start of injection of rocuronium to no response to TOF stimulation (TOF count = 0); clinical duration defined as the time from start of injection of rocuronium to the return of the 4^th^ response of the TOF (TOF count = 4) and recovery to the TOF-ratio of 0.9 and 1.0, defined as the time from start of injection of rocuronium to a TOF ratio above 0.9 and 1.0, respectively. For each time interval, the first of three consecutive corresponding values were considered. In addition, the deepest level of neuromuscular block during onset was also quantified.

### Endpoints

Recovery to a TOF ratio ≥ 0.9 was the primary endpoint of this study. Secondary outcomes were onset time, maximum depth of neuromuscular block, clinical duration and recovery to a TOF ratio ≥ 1.0.

### Statistical analysis

A sample size estimation was done to detect a difference of at least 30% in the recovery to a TOF ratio ≥ 90%. Under intravenous anesthesia mean time to a TOF ratio recovery ≥ 0.9 for adult patients is 60 min with a standard deviation of 11 min [15]. With an alpha risk of 5% and a power of 90%, 14 patients needed to be included in each group. In order to compensate for possible dropouts, 35 patients were included in total.

When continuous variables were compared between 2 groups, the assumptions in the t-test, i.e. homogeneity of variances and normality of residuals were tested by the Bartlett’s test of homogeneity of variances and the Shapiro-Wilks test for the normality of the residuals. If the underlying assumptions were met, a t-test was performed, and data are presented as mean ± standard deviation; if not, a nonparametric Wilcoxon rank test was used and data are presented as median and interquartile range. Discrete variables were compared using a Pearson Chi-square test. When paired differences were tested for continuous variables, a t-test for paired data was used on variables for which a normal distribution is observed for the difference between the two variables and data are presented as mean ± standard deviation. Otherwise, a Wilcoxon test for paired data was used and median and inter-quartile range are presented.

## Results

Thirty five patients were enrolled between May 2020 and March 2021. Data from 3 patients had to be excluded from analysis because of study protocol violation: in 2 patients anesthesia was maintained with sevoflurane instead of propofol and 1 patient received an initial bolus of rocuronium 0.9 mg/kg. Data from 32 patients were complete and could be analyzed.

Of the 32 patients included 16 were ≥ 80 years and 16 were between 20–50 years old. Patients in the group 80^+^ were 85 years old [82–88] and patients in the group 20–50 were 39 years old [28–42]. The female/male ratio was 4/12 in the group 80^+^ and 6/10 in the group 20–50. The weight of patients in group 80^+^ was 70.5 ± 14.0 kg and the BMI was 25.3 ± 3.9. The corresponding values in the group 20–50 were 69.6 ± 10 kg and 24.2 ± 3.9, respectively. Elderly patients had more comorbidities and a higher ASA score than the younger patients (Table 1).

**Table 1:** Demographic data

	Elderly n = 16	Young Adults n = 16	*P* value
Age, years	85 [82-88]	39 [28-42]	< 0.001
Sex (female/male)	4/12	6/10	0.70
Weight, kg	70.5 ± 14	69.6 ± 10	0.83
Height, cm	167 ± 10	170 ± 8	0.33
BMI, kg/m^2^	25.3 ± 3.9	24.2 ± 3.9	0.45
ASA	3 [2-3]	2 [1-2]	< 0.001
Comorbidities			
Cardiovascular	16/16 (100%)	0/16 (0%)	< 0.001
Renal	7/17 (44%)	0/16 (0%)	0.01
Hepatic	0/16 (0%)	0/16 (0%)	-
Diabetes	5/16 (31%)	2/16 (13%)	0.39

Values are expressed either as median [percentile 25-percentile75], mean ± SD or N (%)

In the group 80^+^ onset time was 190 ± 46 s and the corresponding value in the group 20–50 was 123 ± 40 s; *P* < 0.001. The deepest level of neuromuscular block detected was PTC 3.5 and PTC 9, respectively; *P* = 0.07.

The clinical duration was 52 [48–69.5] min in the group 80^+^. The corresponding values in the group 20–50 was 36 [34–41] min, respectively; *P* < 0.001.

Recovery to a TOF-ratio ≥ 0.9 occurred after 77.5 [71–88.5] min and a TOF-ratio ≥ 1.0 was reached after 91.2 [82.2–98] min in the group 80^+^. The corresponding values in the group 20–50 were 53.5 [49.5–55.5] min and 59.5 [56.5–70.25] min, respectively; *P* < 0.001.

## Discussion

The most important findings of the present study were: firstly, both clinical duration and duration until 90% and 100% recovery are significantly prolonged in the group 80^+^. Secondly, compared to younger adults the onset of neuromuscular block is significantly slower in the group 80^+^. Moreover, a tendency toward deeper maximum levels of neuromuscular block could be observed in group 80^+^ (Table 2). Thus, a shift from rocuronium as a rapid onset, intermediate acting compound to a slower onset and long-acting compound can be observed in patients ≥ 80 years old.

**Table 2:** Pharmacodynamic data

	Elderly n = 16	Young Adults n = 16	*P* value
Onset time, s	190 ± 46	123 ± 40	< 0.001
Maximum depth, PTC	3.5 [0-7.25]	9 [3-10]	0.07
Clinical duration, min	52 [48–69.5]	36 [34-41]	< 0.001
Time to TOF-R 0.9, min	77.5 [71–88.5]	53.5 [49–55.5]	< 0.001
Time to TOF-R 1.0, min	91.2 [82.2–98]	59.5 [56.5–70.25]	< 0.001

*PTC* Post-tetanic count, *TOF-R* Train-of-four ratio

Values are expressed either as median [percentile 25-percentile75], mean ± SD

The significance of an acceleromyographic TOF ratio of 0.9 as a criterion for sufficient neuromuscular recovery has been questioned by Capron et al., proposing recovery of the TOF ratio to 1.0 as benchmark when acceleromyography is used [16]. Recent observations by Blobner et al. confirmed this limitation of an acceleromyographic TOF ratio of 0.9 for clinical decision-making [17]. Hence, in the present study both parameters were assessed: a TOF ratio recovery to 0.9 facilitating comparison with data in the literature, and a TOF recovery to 1.0 indicating acceptable neuromuscular recovery when using acceleromyography. In the present study time course neuromuscular block was assessed with the TOFscan monitor. This AMG device has recently been approved for research purpose [14]. This monitor does not need to be calibrated, as it operates with an alternative option to ensure a constant maximum stimulus, it measures the impedance of the skin directly (resistance) and as long as the resistance of the skin is within the measurement window, the stimulation of the nerve is assured with the user-selected electrical current [18]. Moreover, according to the available evidence normalization of TOF values is not mandatory with this device [14]. Thus, recovery parameters indicated in this study are raw TOF-ratios.

Murphy et al. reported a twice as high incidence of postoperative residual neuromuscular block in geriatric patients compared to younger patients, despite similar neuromuscular management [8]. This increased incidence of residual paralysis in the elderly was associated with a higher incidence of hypoxic events, airway obstruction, and postoperative pulmonary complications. Moreover, even PACU and hospital length of stay was increased in the elderly. Hårdemark Cedborg et al. could report a profound negative impact of residual paralysis on airway integrity in elderly individuals, increasing the incidence of pharyngeal dysfunction from 37 to 71%, with impaired ability to protect the airway [10]. Hence, elderly patients are prone to adverse pharyngeal effects of residual neuromuscular blockade and these effects may further increase the risk of postoperative pulmonary complications in this population segment. The present study gives new insights in neuromuscular block characteristics in the oldest old. Clinical duration and time needed to recover to a TOF-ratio of 90% and 100% of baseline are increased about approximatively 60% compared to the group 20–50. Thus, rocuronium shifts from an intermediate-acting compound to a long-acting compound in patients ≥ 80 years. Therefore, strategies to prevent residual paralysis in this patient population needs to be revised and the poor tolerance of even small degrees of residual paralysis in the elderly should be considered in the context.

Neuromuscular monitoring and pharmacological reversal are key elements in any strategy to prevent postoperative residual paralysis [4]. Thilen et al. recently tested a protocol for the prevention of rocuronium-induced residual neuromuscular block based on qualitative monitoring and an optimized protocol for neostigmine-induced reversal [19]. Timing and dosing of neostigmine were founded on best available evidence in their protocol [20–22]. Basically, a TOF count of 4 at the adductor pollicis was required before starting reversal with neostigmine, 40 μg/kg neostigmine were given at a TOF count of 4 with fade, and a delay of at least 10 min between neostigmine administration and extubation was respected [20–22]. Compared to the pre-protocol practice, the incidence of residual paralysis could be reduced from initially 58% to 35% of patients and severe residual paralysis corresponding to a TOF-ratio < 0.7 could be avoided with this protocol. However, small but clinically relevant degrees of residual paralysis corresponding to a TOF-ratio of 0.7—0.9 remained unchanged with this best practice protocol, suggesting that the association of qualitative monitoring and neostigmine-based reversal has limitations [19]. Findings from Martinez-Ubieto et al. further confirmed this limitation of neostigmine [23]. A lesser relative potency of neostigmine in the elderly further confirmed this assumption and emphasizes the need for age-appropriate reversal strategies for elderly patients [24]. Of interest in this context, McDonagh et al. observed a rapid and complete reversal of rocuronium neuromuscular block in the elderly when sugammadex was given at a TOF count of 2. Sugammadex doses were similar to nonelderly adults although reversal was slightly slower in the elderly cohort: 2.2 min in patients < 65 years, 2.6 min in patients 65–74 years old and 3.6 min in patients ≥ 75 years [25].

Succinylcholine may be contraindicated in many elderly patients because of comorbidities such as renal insufficiency with increased serum potassium levels or hemiplegia to name only a few. That’s why rocuronium may be an interesting alternative for rapid sequence induction (RSI) in the elderly. In the present study, a tendency to deeper maximum level of neuromuscular block could be observed in the elderly (Table 2). However, the study was not primarily designed to detect differences in the depth of neuromuscular block. Moreover, the findings of the present study confirmed previous results reporting a slower onset of rocuronium 0.6 mg/kg in the elderly [25]. In the light of these findings one may question whether rocuronium is still a useful compound for RSI in the elderly. In a cohort of elderly patients between 65–92 years Takagi et al. observed an onset time of 187 s after rocuronium 0.6 mg/kg and increasing the dose of rocuronium to 1 mg/kg decreased the onset time to 104 s [26]. Unfortunately, their study design did not allow to draw conclusions on intubating conditions for RSI after rocuronium 1.0 mg/kg. The slower onset on rocuronium neuromuscular block observed in the study by Takagi et al. may be explained by findings from Shiraishi et al. reporting an inverse relationship between cardiac output and rocuronium onset time in the elderly [27]. However, whether doses of rocuronium higher than 1 mg/kg will further decrease its onset time in the elderly is currently unknown, as is the question what level of neuromuscular block is really required in elderly patients to achieve good to excellent intubating conditions after 45–60 s [28].

### Limits and further research

The new insights in neuromuscular block characteristics in patients ≥ 80 given in this study should incite future research addressing the question of safe management of RSI with rocuronium in the elderly. Especially the optimal rocuronium dose and the required level of neuromuscular block to achieve adequate intubating conditions within 45 to 60 s need to be determined. Moreover, as the present study was designed to assess the impact of increasing age on the pharmacodynamic properties of rocuronium, it does not allow detailed insights in the underlaying mechanisms. This has to be addressed in future studies. To this end patients ≥ 80 with hepatic and/or renal insufficiency should be compared with patients in the same age group but without these organ dysfunctions. In addition, different dosing strategies based on real body weight, ideal body weight, or lean body weight should be evaluated in patients ≥ 80 years, also. Finally, the findings of the present study are limited to an intravenous anesthesia background. Both parameters, duration and recovery of rocuronium may be significantly delayed during volatile anesthesia compared to TIVA, as volatile anesthesia may potentiate the effect of nondepolarizing neuromuscular blocking agents.

In conclusion, the present study observed a shift from rocuronium as a rapid onset and intermediate acting compound in the group of younger adults to a slower onset and long-acting compound patients ≥ 80 years old. Age-appropriate strategies for the management of neuromuscular blockade are required for the oldest old.

## Data Availability

The datasets used and analyzed during the current study are available from the corresponding author on reasonable request.
